# A Comparative Study of Laparoscopic Skills Between Novices and Experts: How to Steepen the Learning Curve

**DOI:** 10.7759/cureus.75069

**Published:** 2024-12-03

**Authors:** Julien J Haber, Elie Helou

**Affiliations:** 1 Urology, Université Saint-Joseph, Hôtel-Dieu de France University Hospital, Beirut, LBN

**Keywords:** experts, knot tying, laparoscopy, learning curve, needle handling, novices

## Abstract

Introduction and aim

Laparoscopic surgery has revolutionized the field of surgery over the past few decades. The learning curve in laparoscopy is known to be slow, flat, and complex. This study aims to conduct a comparative analysis of laparoscopic skills, specifically focusing on suturing, knot tying, and needle handling, between novices and experts. The purpose is to objectively quantify the disparities in skill proficiency, identify specific areas needing improvement in training curricula, and contribute to the development of more effective training methodologies for emerging laparoscopic surgeons.

Methods

Residents from different specialties and institutions had their laparoscopic training and evaluation sessions recorded during their curriculum and compared with the performance of experienced surgeons from the Hôtel-Dieu de France University Hospital (Beirut, LBN) during live surgeries. This comparative study was based on the universally recognized Global Operative Assessment of Laparoscopic Skills (GOALS) score and an assessment of a detailed set of laparoscopic skills and techniques used during needle handling and knot tying.

Results

Twenty-one tasks performed by novices and 11 tasks performed by experts were considered. A significant difference was found in the GOALS score between the two groups (experts: 23.4/25; novices: 15.9/25). Moreover, a statistically significant difference was found to be present in favor of the experts in the following skills/techniques: using thread handling and forceps rotation for needle manipulation, laying the needle on a fixed driver arm before grasping it, using needle curvature for knot tying, using upward-facing forceps convexity when tying, using an open thread loop before tying, thread handling capacity, knot tying capacity, and number of needle skills performed per task.

Conclusion

This study demonstrates that many micro-steps in laparoscopic suturing are more prevalent among expert surgeons than among trainees. Incorporating these micro-steps into training could significantly accelerate learning curves, enabling trainees to refine their skills more efficiently and keep pace with the latest surgical advancements in their specialties.

## Introduction

Laparoscopic surgery, a pivotal advancement in minimally invasive surgical techniques, has revolutionized the field of surgery over the past few decades. Characterized by small incisions and the use of a camera and specialized instruments, laparoscopy offers numerous benefits over traditional open surgery, including reduced recovery time and minimal scarring. However, the complexity and finesse required for expertise in laparoscopic procedures underscore the importance of mastering specific skills such as suturing, knot tying, and needle handling. These skills are fundamental to the safety and efficacy of laparoscopic interventions and present unique challenges due to the constraints of limited movement and indirect visualization.

The proficiency in laparoscopic skills varies significantly between novice surgeons, often residents in the early stages of their surgical training, and expert surgeons, who have years of hands-on experience in the operating room. This variance is not only a reflection of experience but also of the training methods and learning curves associated with acquiring such complex skills.

The concept of a learning curve was first described by Ebbinhaus in 1885 [1] as the retention of memorized information and was put into medical practice in 1980 [2]. It is a graphic representation of how many times a certain procedure needs to be repeated before the skills necessary to perform it adequately and safely are acquired [3]. The learning curve in laparoscopy is known to be slow, tough, and complex due to the various cognitive skills required (hand-eye coordination, adaptation to loss of third dimension, fulcrum effect, etc.), and mastery of laparoscopic skills cannot merely rely on a ‘trial and error’ concept [4]. Moreover, with the advent of robotic surgery and the known and proven transfer of skills from laparoscopy to robot-assisted surgery [5], expectations are becoming higher on the surgical resident and trainee to steepen his/her learning curve to become a trusted and recognized practitioner [6].

This study aims to conduct a comparative analysis of laparoscopic skills, especially suturing, knot tying, and needle handling, between novices and experts. Understanding the differences in skill levels between these two groups is crucial for optimizing surgical training programs and ensuring patient safety. By utilizing a combination of video analysis from both training simulations and real surgeries, this research seeks to objectively quantify the disparities in skill proficiency, identify specific areas needing improvement in training curricula, and contribute to the development of more effective training methodologies for emerging laparoscopic surgeons. This study not only addresses a critical gap in the existing medical literature but also holds significant implications for surgical education and patient outcomes in laparoscopic surgery. The abstract of this study article was presented at the annual research meeting of Université Saint-Joseph (Beirut, LBN) on June 7, 2024.

## Materials and methods

For this comparative study, 10 residents (including the author) from different specialties (urology, general surgery, and gynecology) and different institutions were enrolled as novices, and their surgical performance was depicted through video recordings of their work, mainly in surgical lab settings. They thus completed different tasks in different contexts throughout their academic year. Those tasks encompassed primarily laparoscopic standard surgical knots, square knots, and running sutures but also involved some tissue dissection and structure ligation. The training material used in the lab setting ranged from inert tissues like polypropylene meshes to ex-vivo tissues like sheep intestines and in-vivo tissues such as live anesthetized pigs. On the other hand, five experienced surgeons from Hôtel-Dieu de France University Hospital (Beirut, LBN) were enrolled as experts. They had their performance recorded during live surgeries on patients, mainly from the urology department (with one surgeon also having a performance recorded in a surgical lab setting).

The same tasks were evaluated among experts and novices, with a focus on suturing and knot tying tasks. The distribution of the tasks among experts and novices is detailed at the beginning of the 'Results' section. Both novices and experts gave their informed consent to the recording of their work. Consent was also obtained from the patients whose laparoscopic surgeries were recorded and used for the study. The performance of these two groups was assessed and graded using the Global Operative Assessment of Laparoscopic Skills (GOALS) system, a widely used, recognized, and validated laparoscopic skill assessment tool [7,8], which is divided into five subsets, each graded out of 5 for a total score out of 25. The GOALS score template is shown in Table 1.

**Table 1:** The GOALS score template Sourced from *A Preliminary Investigation of General and Technique-specific Assessments for the Evaluation of Laparoscopic Technical Skills* by Vergis et al. [9] distributed under the terms of the Creative Commons Attribution License CC-BY 3.0. GOALS: Global Operative Assessment of Laparoscopic Skills

Criteria	1	2	3	4	5
Depth perception	Constantly overshoots target, wide swings, slow to correct		Some overshooting or missing target, but quick to correct		Accurately directs instruments in the correct plane to target
Bimanual dexterity	Uses only one hand, ignores non-dominant hand, poor coordination between hands		Uses both hands, but does not optimize interaction between hands		Expertly uses both hands in a complementary manner to provide optimal exposure
Efficiency	Uncertain, inefficient efforts, many tentative movements, constantly changing focus or persisting without progress		Slow, but planned movements are reasonably organized		Confident, efficient, and safe conduct, maintains focus on task until it is better performed by way of an alternative approach
Tissue handling	Rough movements, tears tissue, injures adjacent structures, poor grasper control, grasper frequently slips		Handles tissue reasonably well, minor trauma to adjacent tissue (i.e., occasional unnecessary bleeding or slipping of the grasper)		Handles tissues well, applies appropriate traction, negligible injury to adjacent structures
Autonomy	Unable to complete entire task, even with verbal guidance		Able to complete task safely with moderate guidance		Able to complete task independently without prompting
Total	/25				

Thus, the components of the GOALS score and the total GOALS score were compared between the novices and the experts, along with the time taken to perform a laparoscopic task, most notably performing a single knot suture or a running suture. Furthermore, different subsets of laparoscopic skills and techniques were compared between the two groups (Table 2).

**Table 2:** Specific needle and knot skills and techniques included in the comparison

Category	Description
Needle skills	Leaning/laying needle on surrounding planes/tissues for manipulation
	Using shearing forces to manipulate the needle
	Relying on thread handling with the needle holder to manipulate the needle
	Using forceps rotation movements to manipulate the needle
	Laying/pressing needle on fixed needle holder arm before firmly grasping it
	Using thread grasping to improve exposition (during a running suture)
Knot type	Standard surgeon’s knot or square knot
Knot skills	Thread length handling capacity
	Knot tying capacity
	Using the needle curvature for knot tying when needed (e.g. short remaining thread)
	Using the forceps in a downward vs. upward facing convexity shape to tie
	Employing a closed/tight around needle holder loop vs. an open/loose around needle holder thread loop to tie

Each of these skills was evaluated and scored as absent (0) or present (1) during the participant’s work, except for the thread length handling capacity and the knot tying capacity, which were graded out of 2, with 0 indicating the inability or failure to manifest the defined capacity, 1 indicating the moderate ability to perform the skill, and 2 meaning the ability to perform the skill with no difficulty. A p-value threshold of 0.05 was used for comparison.

## Results

A total of 32 performed tasks were recorded in the comparative study, divided into 21 tasks performed by novices (66%) and 11 tasks performed by experts (34%). The p-value of the normality test was < 0.05, meaning the population studied followed the normal distribution. The single knot was performed in 27 of those 32 tasks (84%); nine times by the experts (33.3%) and 18 times by the novices (66.7%). A running suture was performed in 16 of the 32 tasks (50%); 10 times by the experts (62.5%) and six times by novices (37.5%). Other tasks were performed and assessed in five out of 32 instances (15.6%), i.e., once in the expert group (20%) and four times in the novice group (80%). Twenty of the 21 tasks (95.2%) performed by the novice group were in a lab setting (10 in the dry lab, five in the wet lab, and five on live pigs), and one task (4.8%) was recorded during a live surgery. Ten out of 11 tasks performed by the expert group (90.9%) were recorded during live surgeries, and one task (8.9%) was recorded in a lab setting (dry lab).

The GOALS components and total score comparison

A statistical difference was found between experts and novices in favor of the experts in four out of the five components of the GOALS score: depth perception, bimanual dexterity, efficiency, and tissue handling, while no difference was found in autonomy. The GOALS score was thus statistically different in favor of the experts (23.4 ± 1.1 out of 25, versus 15.9 ± 2.9 out of 25 for the novices; p-value 0.000). The time for single knot and running suture completion wasn’t significantly different between the two groups. The aforementioned results are represented in Table 3.

**Table 3:** The GOALS components and total score comparison GOALS: Global Operative Assessment of Laparoscopic Skills

Criteria	Average ± Standard deviation (SD)		p-value (Student's t-test)
	Experts	Novices	
Depth perception	4.7±0.5	3.1±0.7	0.000
Bimanual dexterity	4.9±0.3	3.0±0.6	0.000
Efficiency	4.4±0.7	2.7±0.9	0.000
Tissue handling	4.4±0.5	3.2±0.9	0.000
Autonomy	5±0.1	3.9±1.2	0.792
GOALS score	23.4±1.1	15.9±2.9	0.000
Time for single knot (seconds)	248±142 (120-540)	216±108 (95-480)	0.577
Time for running suture (seconds)	1084±856 (300-3000)	660±289(450-990)	0.212

Specific skills/techniques comparison

A significant difference was found in favor of the experts in the following specific needle skills: relying on thread handling with the needle holder to manipulate the needle, using forceps rotation movements to manipulate the needle, and laying or pressing the needle on the fixed needle holder arm before firmly grasping it. Additionally, some knot skills were notably superior or significantly more prevalent among the experts, such as thread length handling capacity, knot tying capacity, using the needle curvature for knot tying when needed, using the forceps in an upward-facing convexity shape to tie, and employing an open or loose around-the-needle-holder thread loop to tie. Conversely, a significant difference in favor of the novices was observed in other knot skills, including using the forceps in a downward-facing convexity shape to tie and employing a closed or tight around-the-needle-holder thread loop to tie.

No significant difference was noted between the two groups for the following skills and techniques (all needle skills): leaning or laying the needle on surrounding planes or tissues for manipulation, using shearing forces to manipulate the needle, and using thread grasping to improve exposition during a running suture. As for the knot type, the standard surgeon’s knot was more frequently performed by the experts, while the square knot was more frequently performed by the novices (significant difference). These results are presented in Table 4.

**Table 4:** Specific techniques and skills comparison NM: Needle manipulation, NH: Needle holder, N/A: Not applicable

Specific skills/techniques	Score	Experts (total = 11 tasks)	Novices (total = 21 tasks)	p-value (two proportions test)
Leaning/laying on surrounding planes/tissues for NM	0	8 (73%)	11(52%)	0.269
	1	2 (18%)	7 (33%)	
	N/A	1 (9%)	3 (14%)	
Use of shearing forces for NM	0	1 (9%)	7 (33%)	0.066
	1	9 (82%)	12 (57%)	
	N/A	1 (9%)	2 (10%)	
Thread handling with needle holder for NM	0	0 (0%)	7 (33%)	0.001
	1	10 (91%)	12 (57%)	
	N/A	1 (9%)	2 (10%)	
Forceps rotation for NM	0	2 (18%)	14 (67%)	0.000
	1	8 (73%)	4 (19%)	
	N/A	1 (9%)	3 (14%)	
Laying the needle on the fixed needle holder hand before grasping	0	3 (27%)	18 (86%)	0.001
	1	7 (64%)	0 (0%)	
	N/A	1 (9%)	3 (14%)	
Using thread grasping for exposition	0	6 (55%)	4 (19%)	0.398
	1	4 (36%)	1 (5%)	
	N/A	1 (9%)	16 (76%)	
Standard surgeon’s knot	0	0 (0%)	12 (57%)	0.000
	1	9 (82%)	7 (33%)	
	N/A	2 (18%)	2 (10%)	
Square knot	0	8 (73%)	6 (29%)	0.000
	1	1 (9%)	13 (62%)	
	N/A	2 (18%)	2 (10%)	
Thread handling capacity/2	0	0 (0%)	0 (0%)	0.000
	1	0 (0%)	13 (62%)	
	2	9 (82%)	6 (29%)	
	N/A	2 (18%)	2 (10%)	
Knot tying capacity/2	0	0 (0%)	0 (0%)	0.000
	1	0 (0%)	8 (38%)	
	2	9 (82%)	11 (52%)	
	N/A	2 (18%)	2 (10%)	
Using needle curvature for knot tying (when needed)	0	0 (0%)	4 (19%)	0.001
	1	4 (36%)	2 (10%)	
	N/A	7 (64%)	15 (71%)	
Forceps convexity facing down during knot tying	0	8 (73%)	0 (0%)	0.000
	1	1 (9%)	19 (90%)	
	N/A	2 (18%)	2 (10%)	
Forceps convexity facing up during knot tying	0	1 (9%)	18 (86%)	0.000
	1	8 (73%)	1 (4%)	
	N/A	2 (18%)	2 (10%)	
Open/loose loop around NH	0	0 (0%)	16 (76%)	0.000
	1	9 (82%)	3 (14%)	
	N/A	2 (18%)	2 (9%)	
Closed/tight loop around NH	0	9 (82%)	3 (14%)	0.000
	1	0 (0%)	16 (76%)	
	N/A	2 (18%)	2 (9%)	

Furthermore, we assessed the difference between the number of total needle skills per task (the first five skills in descending order in Table 2: (1) leaning/laying the needle on surrounding planes/tissues for manipulation, (2) using shearing forces to manipulate the needle, (3) relying on thread handling with the needle holder to manipulate the needle, (4) using forceps rotation movements to manipulate the needle, and (5) laying/pressing the needle on the fixed needle holder arm before firmly grasping it), applied by each participant of the two groups. The results were as follows: 3 ± 1 needle skills per task for the experts and 2 ± 1 needle skills per task for the novices. After comparison using the Student's t-test, a statistical difference was found with a p-value of 0.018.

## Discussion

Minimally invasive surgical procedures have a flat learning curve, especially in the initial phase of a surgeon's training [10]. The ubiquitous law of ‘practice makes perfect’ has long been investigated, especially in laparoscopic surgery [11]. The learning curve in laparoscopy is known to be flat for beginners [12] and has been a subject of discussion ever since the introduction of that surgical modality [13]. Many studies have been made to assess the needs of residents’ training and improve their learning curve. Gozen and Akin studied the positive impact of well-designed and structured training programs and curricula on laparoscopy training [14]. Spille et al. acknowledged the effect of the pelvitrainer in the learning process [15]. Enani et al. demonstrated the importance of incorporating cognitive knowledge, motor memory, choreography, and technical step learning in laparoscopic training [16].

Differences shown in our study between experts and novices concerning depth perception, bimanual dexterity, efficiency, and total GOALS score underline the importance of incorporating training tasks addressing those specific skills in training programs worldwide, as is done in the institution where the current study was performed. Other comparative studies have been made in the field of minimally-invasive surgery learning, exploring objective differences between novices and experts, like hand motion [17] or evaluating the difference between the two sides in the crossover of skills from laparoscopic to robotic surgery [18,19]. Likewise, many evaluative methods, models, instruments, and devices have been studied [20,21] to objectify and improve trainees’ performance evaluation. However, the exclusivity of this study lies in the analysis of each micro-step performed during the laparoscopic suture realization. To our current knowledge, no study yet has dived into the specific steps of a laparoscopic task, mainly the laparoscopic suture, by dissecting it to find the differences in techniques and skill sets used by the operator and comparing the consequent findings between experts and novices.

The largely non-significant difference in leaning/laying on surrounding planes/tissues for needle manipulation with a relatively low rate of usage by both novices and experts could be explained by a reluctance or unwillingness of both sides to consistently apply this technique, as it can lead to damage to surrounding tissues/planes if excessive force is used. The use of shearing forces to manipulate the needle was non-significantly higher (p = 0.066) in the novice group, as this seems like a more intuitive way to handle the needle. However, several expert surgeons used this technique, although slightly less frequently but more efficiently towards a correct needle orientation. This demonstrates a kind of reluctance of the novices to perform other, more reproducible techniques for needle orientation, like thread handling, which was statistically more frequent among experts. It is worth noting that thread handling for needle orientation was the main technique taught and explained to residents before starting their laparoscopic training. The forceps rotation difference also translates to a lack of awareness of the residents that such movements can significantly improve efficiency and needle manipulation capacity.

Laying/pressing the needle on the fixed needle holder arm before firmly grasping it was absent in the novice group, and comparing this skill showed a significant difference in favor of the experts. This skill is a small but significant detail in needle manipulation, which can be learned over time. Consequently, implementing it and insisting on its application may prove crucial to laparoscopic skill and efficiency improvement. We also demonstrated the fact that surgeons use more needle techniques per task than novices, which implies a better versatility among surgeons that enables them to use different techniques, according to each surgical requirement, to achieve the desired goal. This versatility could prove to be a necessary advanced skill to acquire during the novices’ later stages of training.

Using thread grasping to improve exposition is an important skill to utilize during a running suture, and residents seem to be aware of it, as no statistical difference was demonstrated between them and the experts. The standard surgeon knot type was more frequently employed by the experts, and the square knot type by the novices, as the latter is the knot type that novices were prompted to utilize at the beginning of their training by their supervisors. Therefore, no conclusions can be made as to the impact of the knot type on laparoscopic skills. The significant difference in thread length handling capacity shows a probable lack of awareness among the novices that correctly managing the thread length can positively influence their task efficiency. Thus, this skill needs to be thoroughly taught and included in corresponding training programs, as well as for knot tying capacity. Using the needle curvature for knot tying when needed was more frequently and efficiently used by experts than novices, which could translate to a lack of knowledge by the novices that this technique can be used to ‘save’ them during tricky situations in their upcoming surgical career (e.g. when the remaining thread length is too small to perform a standard knot using thread manipulation only).

Novices tend to orient the curved forceps in a downward-facing convexity, whereas the experts use an upward-facing forceps convexity much more often. Additionally, novices tighten the thread with their forceps around the needle holder during suture finishing, whereas surgeons more frequently employ an open, loose loop. The teaching of this set of skills also proves to be important in improving the knot tying efficiency among novices.

Limitations

This study was performed on a relatively small number of participants (10 novices performing 21 tasks and five experts performing 11 tasks; a total of 15 participants performing 32 tasks), which can render generalization of the results somewhat tricky. The novices’ performances were recorded at different times during their academic year, which might constitute a bias since their performance significantly improved from the beginning to the end of their training, and this improvement was not taken into consideration during data analysis. Moreover, only one novice performance was recorded during a live surgery task, which didn’t involve a laparoscopic suture. All the other novice tasks were performed in a lab setting, while the experts’ tasks were conducted in a live surgery setting, with surgeries sometimes comprising a high level of task difficulty. This could have significantly impacted the time needed to perform a certain task, which showed no statistically significant difference between novices and experts, mainly because live surgical tasks are more difficult, pressure-generating, and time-consuming than lab tasks. Another limitation could be the difference in laparoscopic instrument quality used by novices during their training compared to the more reliable instruments used in live surgeries.

## Conclusions

The learning curve in laparoscopy is notably flat and slow, particularly at the onset of residency training. Simultaneously, expectations and requirements are increasing due to advancements in surgical techniques across various fields, namely the advent of robotic surgery. Trainees now have less time to learn but more techniques and surgical modalities to master to become proficient surgeons. Our study demonstrates that many micro-steps in laparoscopic suturing are more prevalent among expert surgeons than among trainees. Incorporating these micro-steps into training could significantly accelerate learning curves, enabling trainees to refine their skills more efficiently and keep pace with the latest surgical advancements in their specialties.
